# Relationship between Hounsfield Unit in CT Scan and Gray Scale in CBCT

**DOI:** 10.5681/joddd.2014.019

**Published:** 2014-06-11

**Authors:** Tahmineh Razi, Mahdi Niknami, Fakhri Alavi Ghazani

**Affiliations:** ^1^Assistant Professor, Department of Oral and Maxillofacial Radiology, Faculty of Dentistry, Tabriz University of Medical Sciences, Tabriz, Iran; ^2^Assistant Professor, Department of Oral and Maxillofacial Radiology, Faculty of Dentistry, Tehran University of Medical Sciences, Tehran, Iran; ^3^Post-graduate Student, Department of Oral and Maxillofacial Radiology, Faculty of Dentistry, Tabriz University of Medical Sciences, Tabriz, Iran

**Keywords:** Gray scale, cone-beam computed tomography (CBCT), computed tomography (CT)

## Abstract

***Background and aims.*** Cone-beam computed tomography (CBCT) is an imaging system which has many advantages over computed tomography (CT). In CT scan, Hounsfield Unit (HU) is proportional to the degree of x-ray attenuation by the tissue. In CBCT, the degree of x-ray attenuation is shown by gray scale (voxel value). The aim of the present study was to investigate the relationship between gray scale in CBCT) and Hounsfield Unit (HU) in CT scan.

***Materials and methods.*** In this descriptive study, the head of a sheep was scanned with 3 CBCT and one medical CT scanner. Gray scales and HUs were detected on images. Reconstructed data were analyzed to investigate relationship between CBCT gray scales and HUs.

***Results.*** A strong correlation between gray scales of CBCT and HUs of CT scan was determined.

***Conclusion.*** Considering the fact that gray scale in CBCT is the criteria in measurement of bone density before implant treatments, it is recommended because of the lower dose and cost compared to CT scan.

## Introduction

Cone-beam computed tomography (CBCT) is an imaging system which has many advantages over computed tomography (CT), including lower radiation dose to the patient, shorter acquisition times, reasonable price and submillimeter resolution.^1^ Beam hardening artifacts, more scattered radiation and inability to show the actual Hounsfield Unit (HU) similar to CT scan can be noted as the disadvantages of CBCT.^1,2^

CBCT can be used to determine bone density and bone quality for dental implant placement, bone height and width, distance to anatomical structures such as the mandibular canal and sinuses, and the stability of the implant.^2-4^

In CT scan, Hounsfield Unit is proportional to the degree of x-ray attenuation and it is allocated to each pixel to show the image that represents the density of the tissue.^1^ In CBCT, the degree of x-ray attenuation is shown by gray scale (voxel value).^4^Although CBCT manufacturers and software providers present gray scales as the HUs, it is important to note that these measurements are not true HUs.^2,5^ Gray scale is used in cases like determining the kind of bone in placing dental implants, pathologic lesions, evaluation of the airways and determining the stability of the implant.^2,4-6^

Although high levels of radiation scatter and artifacts in CBCT have been reported as the disadvantages of CBCT in the estimation of bone density, a large number of studies have shown a linear relationship between HU in CT scan and gray scale in CBCT and suggested that voxel value in CBCT can be used for estimation of bone density.^2,4,5^

The results of a study by Mah indicated a strong linear relationship between the gray scales ​​in CBCT and HU in CT. In this study, a phantom containing tissue-equivalent material with homogeneous density of the material structure was used.^2^ Strong linear relationships were obtained from a study by Jayasanker, in which a bone-equivalent material was used.^4^

Similar results were obtained in another study carried out on human cadaver mandibular bone.^11^However, since there is no actual Hounsfield Unit in CBCT, and given that phantoms in previous studies have been mostly used with tissue-equivalent densities of homogeneous materials throughout the whole structure, the results cannot be generalized to clinical applications with non-homogeneous human tissues. In a study by Parsa on human cadaver mandibles, soft tissues such as the ones around spine and tongue were excluded and only the hard tissues were studied, which were not generalized to physiological tissues, either. With regard to the importance of the clinical application of gray scale, for example, in determining the bone quality for dental implant placement and the increasing use of CBCT for dental applications, the present study was undertaken to use normal tissues in this study. In addition, the CBCTs reviewed in this study differed from the ones in some previous studies.

## Materials and Methods

A descriptive study was designed to investigate the relationship between gray scale in cone-beam CT (CBCT) and Hounsfield Units (HU) in CT scan. The head of a sheep was used a day after killing and all the imaging techniques were carried out on the same day.^7,8^ To avoid damage to hard and soft tissues, the sample was stored and transported at 4°C.

The studied tissues were cortical bone, cancellous bone, muscle, fat, cartilage, enamel, dentin and the sinus area.

First, an initial head scan was carried out to rule out any lesion and fracture. The first scanning procedure was carried out using NewTom VG (Verona, Italy), which is a cone-beam x-ray machine with 0.3-mm voxel size and rotation of 360°, and scanning was carried out at 110 kVp, 4.71 mA and 3.6 s. Primary and final reconstructions were carried out by NNT Viewer version 2.21.

The next scanning was carried out by Planmeca Promax 3D (Helsinki, Finland), which is a device with cone-shaped x-ray, flat panel detector, rotation of 270° and a voxel size of 160 µm; scanning was carried out at 16 mA and 84 kVp.

Planmeca Romexis 2.3.1 software was used for primary and final reconstructions.

The subsequent scan was carried out by Scanora Soredex 3DX CBCT, which is a cone-beam x-ray machine with a voxel size of 0.35 mm and rotation of 360° and the scan was carried out at 13 mA and 90 kVp. On-demand 3D (Cybermed) software was used for primary and final reconstructions.

The final scan was carried out by Somatom Sensation CT scan (Siemens, Germany). The scans were carried out using the head and sinus program at 110 kVp and 110 mA. The studied images were axial sections. The images were examined by the special software for each device to obtain the gray scale.

In order to determine the gray scale, a square of 10×10 pixel was considered as a region of interest (ROI) in the center of every tissue and the gray scale of that area was obtained. The scroll bar was moved until reaching the center of the tissue. In the tongue muscle, for example, in sagittal views the images were moved forward and backward, in coronal section to the left and right, and in the axial section they were moved up and down to reach the center of tissue. Thus, all the evaluations were carried out on the same location for all the machines.

In CT, HU was obtained from the CT scan device in a similar manner.^9^

A 17-inch cathode ray tube (CRT) desktop monitor, Hansol Ep, Iran, 32720 bits, 256 colors, 768×1024 resolution, was used in a room with reduced ambient light to display the images.

## Results

SPSS 19 was used to calculate the means and the standard deviations. The linear relationship equation was used to show the relationship between gray scale and HU.

The means of gray scales in NewTom VG, Scanora Soredex and Planmeca were 616±373, 619±353, 633±341, respectively, and the average of CT HU in Somatom CT scan was 685±407. T-test did not show any significant differences between the gray scale of each CBCT and CT HU (P=0.001).

**Figure 1.**
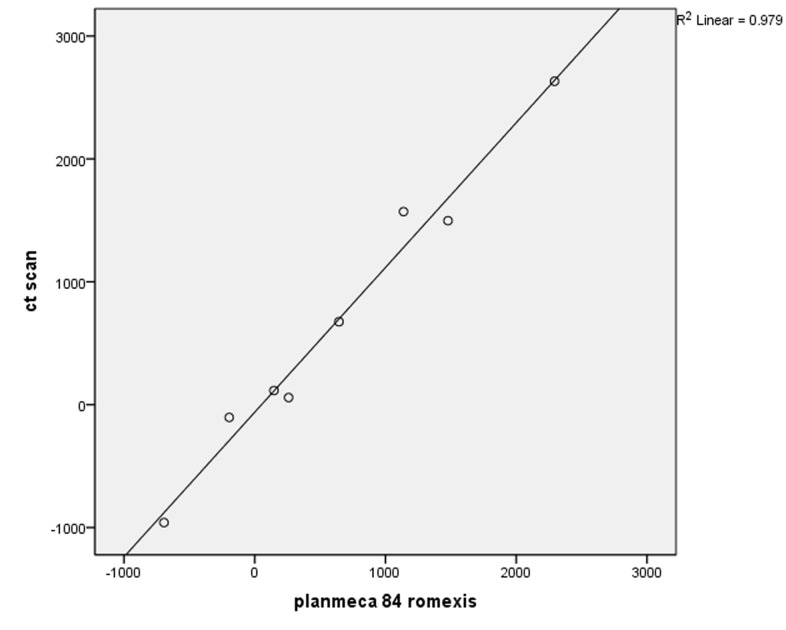
Linear regression of the results from the NewTom VG.

**Figure 2.**
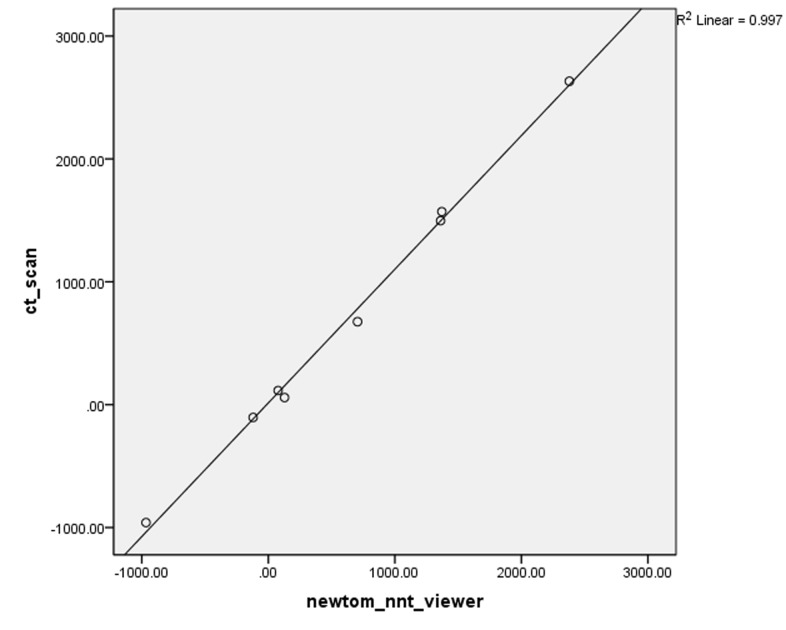
Linear regression of the results from the Scanora Soredex.

**Figure 3.**
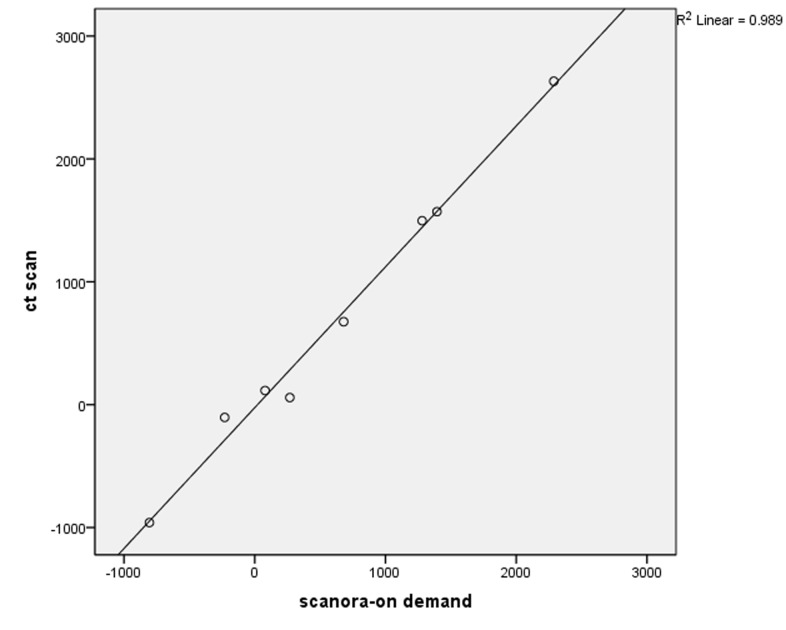
Linear regression of the results from the Planmeca Promax.

In Newtom VG, R^2^ was calculated at 0.997 and the equation of the regression was HU = 14.621 + 1.088 × gray scale (Figure 1). In Scanora Soredex, R^2^ was estimated at 0.989 and the equation of the regression was HU = −24.052 + 1.146 × gray scale and the relationship was linear (Figure 2). In Planmeca, R^2^ = 0.979 and the equation of the regression was HU = −61.098 + 1.178 × gray scale (Figure 3).

There was a linear relationship between gray scale and CT HU in all the three CBCTs.

## Discussion

Diagnosis of changes in bone density and hard tissues is important in radiographic images because changes outside the normal range may indicate disease; early diagnosis is important for the patient^’^s health. The diagnosis of density changes in all the common extraoral radiographic techniques is based on the darkness and brightness of images, expressed with Hounsfield Unit in CT scan and with gray scale in CBCT.

In a study in which images were taken with NewTom 3G, CBCT showed that the gray scale was positive in the solid lesion and negative in fluid and air-filled lesions. Thus, CBCT can help in the differential diagnosis of these lesions.^10^

Gray scale has also been effective in determining the amount of bone density after periapical lesion treatment.^11^

Gray scale has many applications in determining the origin of lesions and tissues and density changes, but gray scales ​​are not the same in various devices. So far, CBCT manufacturers have not introduced a standard system for displaying gray scale.^2,12^

HU is a standard scheme for measuring CT values​​ in CT scan. Some studies have shown a strong linear relationship between HU and gray scale. However, gray scale is different due to higher noise levels, more scattered radiation, high heel effect and beam hardening artifacts.^2,4-5^

Mah showed a relationship between HU and gray scale through a linear equation using scans from different materials. However, it was noted that because of the homogeneity of tissue-equivalent material, a study on the living tissue was required.^2^ In this study HU and gray scale were compared in physiological structures. The results showed a linear relationship between HU and gray scales, confirming the findings of Mah on the tissue-equivalent material. In the present study, the obtained values ​​of R^2^ = 0.997 were similar to those of a study by Mah. Although there were differences between the results of various devices, there were no statistically significant differences in the means of gray scales, which probably showed that the similarity of the effective features influenced the gray scale in the three studied systems. In Parsa’s study, the correlation coefficient between HU and gray scale was R^2^ = 0.96. The difference in correlation coefficients might be related to the materials under study and the type of the device. In that study, a dry human mandible was used so that the density changes seemed relatively normal and the gray scales were different from the actual ones. According to the results of various studies, using dry mandible to remove the effect of adjacent tissues, such as the tongue and spinal tissues, can lead to interferences in determining the gray scale tissues.^5,13^In the present study, hard tissues along with the soft ones were reviewed and the device used in the study was the newer version of the previous study.

In a study carried out by Mah and Jayasankar, despite studying similar materials, the high correlation coefficient results could be related to the lack of soft tissue effect on the results. In the study by Mah, the phantom was placed in water.^2^Jayasankar used acrylic resin as soft tissue-equivalent material and despite the numerical differences between HU and gray scale a correlation coefficient of 0.99 was obtained.^10^

In the present study, there was a similarly strong linear relationship between the gray scale and HU values in all the systems, which can be attributed to similarity of effective factors influencing the gray scale and improvement of the image in the new version of devices.

It is suggested that further studies be carried out with other CBCT scanners used for clinical applications.

## Conclusion

Considering the fact that gray scale in CBCT is the standard for measuring bone density before implant treatment, it is recommended because of the lower dose and cost compared to CT scan.
